# Variant rs17619600 in the gene encoding serotonin receptor 2B (*HTR2B*) increases the risk of gestational diabetes mellitus: a case–control study

**DOI:** 10.1186/s40001-023-01211-6

**Published:** 2023-07-21

**Authors:** Juliana Regina Chamlian Zucare Penno, Daniele Pereira Santos-Bezerra, Ana Mercedes Cavaleiro, Ana Maria da Silva Sousa, Tatiana Assunção Zaccara, Rafaela Alkmin da Costa, Rossana Pulcineli Vieira Francisco, Maria Lucia Correa-Giannella

**Affiliations:** 1 Laboratório de Carboidratos e Radioimunoensaios (LIM-18), Hospital das Clínicas HCFMUSP, Universidade de São Paulo, Av. Dr. Arnaldo 455, Sala #3321, CEP, 01246-000 Sao Paulo, SP Brazil; 2 Departamento de Fisiologia e Biofísica, Instituto de Ciências Biomédicas, Universidade de São Paulo, Av. Prof. Lineu Prestes 1524, Sao Paulo, SP Brazil; 3 Disciplina de Obstetrícia, Departamento de Obstetrícia e Ginecologia, Faculdade de Medicina FMUSP, Universidade de São Paulo, Instituto Central–Hospital das Clínicas, Av. Dr. Enéas de Carvalho Aguiar 255, 10º Andar, Sala 10.093, CEP, 05403-000 Sao Paulo, SP Brazil

**Keywords:** HTR2B, Single nucleotide polymorphisms, Serotonin, Beta cell

## Abstract

**Background:**

During pregnancy, the increase in maternal insulin resistance is compensated by hyperplasia and increased function of maternal pancreatic beta cells; the failure of this compensatory mechanism is associated with gestational diabetes mellitus (GDM). Serotonin participates in beta cell adaptation, acting downstream of the prolactin pathway; the blocking of serotonin receptor B (HTR2B) signaling in pregnant mice impaired beta cell expansion and caused glucose intolerance. Thus, given the importance of the serotoninergic system for the adaptation of beta cells to the increased insulin demand during pregnancy, we hypothesized that genetic variants (single nucleotide polymorphisms [SNPs]) in the gene encoding HTR2B could influence the risk of developing GDM.

**Methods:**

This was a case–control study. Five SNPs (rs4973377, rs765458, rs10187149, rs10194776, and s17619600) in *HTR2B* were genotyped by real-time polymerase chain reaction in 453 women with GDM and in 443 pregnant women without GDM.

**Results:**

Only the minor allele C of SNP rs17619600 conferred an increased risk for GDM in the codominant model (odds ratio [OR] 2.15; 95% confidence interval [CI] 1.53–3.09; *P* < 0.0001) and in the rare dominant model (OR 2.32; CI 1.61–3.37; *P* < 0.0001). No associations were found between the SNPs and insulin use, maternal weight gain, newborn weight, or the result of postpartum oral glucose tolerance test (OGTT). In the overall population, carriers of the XC genotype (rare dominant model) presented a higher area under the curve (AUC) of plasma glucose during the OGTT, performed for diagnostic purposes, compared with carriers of the TT genotype of rs17619600.

**Conclusions:**

SNP rs17619600 in the *HTR2B* gene influences glucose homeostasis, probably affecting insulin release, and the presence of the minor allele C was associated with a higher risk of GDM.

## Introduction

To maintain glycemic homeostasis during pregnancy, the increase in maternal insulin resistance is compensated by hyperplasia and increased function of maternal pancreatic beta cell. The failure of this compensatory mechanism is associated with gestational diabetes mellitus (GDM) [1].

GDM occurs in 10–18% of all pregnancies [2–4] and it confers a higher risk of different pregnancy outcomes as cesarean delivery and possible pregnancy complications for both mother and newborn, such as polyhydramnios, preeclampsia, jaundice, macrosomia, and neonatal hypoglycemia. GDM is also considered a risk factor for type 2 diabetes mellitus (T2D), obesity and cardiovascular disease [5, 6].

Studies conducted in vitro and in rodent models have shown modified expression of many islet genes during pregnancy. Among the most significantly upregulated genes are the ones encoding the two isoforms of tryptophan hydroxylase (*Tph1* and *Tph2*), the rate-limiting enzyme of serotonin (5-hydroxytryptamine, 5-HT) synthesis [7, 8], together with other genes involved in cell cycle regulation and islet regeneration, such as the genes encoding regenerating islet-derived 3 alpha and 3 beta [7]. Βeta cells have the ability to synthesize, store and secrete 5-HT and islet 5-HT content increases during pregnancy [7–9], secondarily to stimulation of TPH1 and TPH2 expression in beta cells. This process is dependent on placental lactogen (PL) acting through prolactin receptors (PRLR) [9]; thus, 5-HT acts downstream of PL signaling to drive beta cell expansion [8, 10, 11].

5-HT receptors are classified into seven different families (HTR1–7), some of which contain different subtypes [12]. All of them, except the HTR3 subtype, are G-protein-coupled receptors which trigger various intracellular signaling pathways; their unique distribution explains the tissue-specificity of serotonin effects [13]. In pregnant mice, the HTR2B expression closely matched the period of increased beta cell proliferation [8, 14] and blocking HTR2B signaling impaired beta cell expansion, causing glucose intolerance [8].

Microarray and ribonucleic acid (RNA) sequencing analyses revealed transcripts of almost all 5-HT receptors in human islets [15, 16] and an in vitro study have shown that the activation of HTR2B promoted glucose-stimulated insulin secretion (GSIS) not only in mouse, but also in human beta cells, suggesting that 5-HT also stimulates insulin release through HTR2B [16].

GDM is a complex condition resulting from environmental and genetic factors. Variants in T2D susceptibility genes involved in insulin secretion (such as *TCF7L2* [coding for transcription factor 7-like 2] and *MTNR1B* [coding for melatonin receptor 1B]) and in insulin resistance (such as *PPARG* [peroxisome proliferator activated receptor γ]) have been associated with GDM, suggesting a similar genetic architecture between these two metabolic diseases, although it is believed that some genes are unique to GDM [17]. Given the importance of the serotoninergic system for the adaptation of beta cells to the increased insulin demand during pregnancy, we hypothesized that genetic variants in the *HTR2B* gene could influence the risk of developing GDM.

## Participants and methods

This was a case–control study that initially recruited 1130 pregnant women between September 2014 and September 2017; 90 refused to participate and 36 were considered ineligible for not meeting the inclusion criteria. Of the 1004 pregnant women then recruited, 108 were lost to follow up, resulting in a final number of 896. All pregnant women were followed-up at the Obstetric Clinic of a tertiary university hospital in the city of São Paulo, Brazil; they were recruited from two outpatient clinics: one specialized in endocrinopathies, where participants with GDM were recruited (*N* = 453) and the other in charge of low-risk pregnancies, where participants without GDM were recruited (*N* = 443). The study was carried out in compliance with the Declaration of Helsinki [18] and was approved by the institutional ethics committees (CAPPesq # 777.904, 09/03/2014). After signing informed consent, participants were evaluated for clinical and biochemical characteristics.

GDM was defined by criteria proposed by the International Association of Diabetes and Pregnancy Study Groups (IADPSG) [19–21] based on first trimester fasting plasma glucose (FPG) ≥ 92 mg/dL (5.1 mmol/L) (*n* = 257) or 2-h OGTT with 75 g of glucose performed between 24 and 28 weeks of gestation with at least one altered value (FPG ≥ 92 mg [5.1 mmol/L], 1-h plasma glucose ≥ 180 mg/dL and 2-h plasma glucose ≥ 153 mg/dL [8.5 mmol/L]) (*n* = 196).

The inclusion criteria for women without GDM were no prior GDM or other metabolic conditions, normal FPG in the first trimester and normal 75 g OGTT between 24- and 28-week gestational age. The inclusion criteria for women with GDM were: GDM diagnosis in the index pregnancy and no use of steroids before GDM diagnosis. For both groups, the exclusion criteria were twin pregnancy, diabetes or known blood glucose alteration before pregnancy, failure to perform OGTT when the first trimester FPG < 92 mg/dL and previous bariatric surgery.

Women with GDM who failed to reach 30% of the glycemic targets (FPG < 95 mg/dL, 1-h postprandial glucose < 140 mg/dL), after dietary and lifestyle modifications, were administered insulin. A 2-h OGTT was performed in the GDM group at 6 to 12-week postpartum, and the American Diabetes Association (ADA) diagnostic criteria for diabetes mellitus (DM) [20] were applied.

### Single nucleotide polymorphisms genotyping

Blood was collected from a peripheral vein at the time of inclusion in the study. In the group without GDM, inclusion occurred after the OGTT performed between 24 and 28 weeks. In the group with GDM, inclusion occurred after confirmation of the diagnosis by FPG or after 24–28 weeks in the participants who were diagnosed by OGTT. Deoxyribonucleic acid extraction from blood leukocytes was carried out by a salting-out procedure [22]. Single nucleotide polymorphisms (SNPs) were genotyped by real-time polymerase chain reaction (StepOne Plus; Applied Biosystems, USA), using predesigned Human TaqMan Genotyping Assays 40X: C__2398885_30 (rs765458), C__30043265_10 (rs10187149), C__32997861_10 (rs17619600), C__27918443_10 (rs4973377), C__29863060_10 (rs10194776). This assay includes two differentially labeled, allele-specific probes (FAM and VIC) and a polymerase chain reaction primer pair that uniquely amplify and provide unmatched specificity for the alleles of interest; allelic discrimination plots were generated for genotype determination (Thermo Fisher Scientific, Waltham, USA). The five Tag SNPs cover approximately 95% of the genetic variability of the extended region of *HTR2B* gene and were selected using a pair wise approach, a r^2^ ≥ 0.8 and a minor allele frequency (MAF) of at least 0.1. The genotyping success rate was ~ 99% for all SNPs. The SNP rs4973377 was not evaluated, because only one genotype was found in the studied population. The Hardy–Weinberg equilibrium (HWE) was tested; the distribution of genotypes was consistent with HWE for all remained SNPs.

### Statistical analyses

The statistical analyses were performed with the use of JMP software version 8.0 (SAS Institute, Cary, NC). Due to non-normal distribution of the data, non-parametric tests were used. Continuous variables are expressed as median and 25–75% interquartile ranges, and categorical variables are expressed as number of cases and percentage of affected individuals. The Mann–Whitney test for independent samples was used to compare continuous variables between the studied groups, while categorical variables were compared by Pearson's *χ*^2^ test, which was also used to compare the frequency of SNPs between the groups.

The HWE was determined using the frequency of alleles in the Pearson's *χ*^2^ test at a significance level of 0.05. The SNPs were evaluated in the rare dominant model and in the codominant model. The magnitude of the risk conferred by the SNPs was estimated using odds ratio (OR) with a 95% confidence interval (CI). To estimate the OR adjusted for potential confounding factors (age at last menstrual period [LMP], previous body mass index [BMI], and weight gain during pregnancy), a binary logistic regression analysis was performed with these factors as covariates in the regression model. The correction for multiple comparisons due to the multiple SNPs tested was made by Bonferroni’s correction, dividing 0.05 by the number of studied SNPs in the *HTR2B* gene. Thus, a *P* < 0.01 (two-tailed) was considered significant.

This study was exploratory in nature. Thus, a convenience sample was used, in which we sought to include as many participants as possible. The power calculation was performed a posteriori using the *GAS Power Calculator* (Genetic Association Study power calculator) [23]. The power of the study was > 90% (100% and 99.7%, respectively) to detect associations of the SNP rs17619600 in the *HTR2B* gene with GDM in the codominant model and in the rare dominant model. Haplotype analysis was performed using the software available online SHEsis. All haplotypes with a frequency < 0.03 were ignored for analysis [24].

The area under the curve (AUC) of plasma glucose during the OGTT was calculated using the trapezoidal method and the results were expressed as mean ± standard deviation. For comparison of the AUC of plasma glucose between genotypes, one-way ANOVA was used, adjusted for age at LMP, previous BMI, and weight gain during pregnancy.

## Results

The characteristics of the pregnant women with and without GDM are shown in Table 1.

**Table 1:** Characteristics of women with and without gestational diabetes mellitus

	Without GDM	With GDM	*P *value
N	443	453	
Age (years)	29.1 (24.4–33.4)	33.2 (28.8–37.1)	< 0.0001
White (%)^a^	85	85	0.94
Parity	1.0 (1.0–2.0)	2.0 (1.0–3.0)	< 0.0001
Pre-pregnancy BMI (kg/m^2^)	24.7 (21.8–28.1)	28.2 (24.8–32.9)	< 0.0001
Positive family history of T2D (%)	49	58	< 0.0001
Weight gain during pregnancy (kg)^b^	12.0 (8.7–15.2)	9.0 (5.0–13.3)	< 0.0001
Preeclampsia (%)	8.4	8.5	0.76
Arterial hypertension (%)	7.5	23.8	< 0.0001
Smoking (%)	9.1	7.9	0.30
Use of medicines (%)	28	51	< 0.0001
Insulin treatment (%)^c^	–	18.7	–
Fasting plasma glucose (mg/dL)	78 (74–82)	92 (83–97)	< 0.0001
Cesarean delivery (%)	48	60	0.001
Newborn birth weight (g)	3240 (2897–3542)	3210 (2780–3512)	0.04

Results expressed as median and interquartile range

BMI: body mass index; GDM: gestational diabetes mellitus; T2D: type 2 diabetes mellitus

^a^Self-defined ethnicity

^b^Weight gain during pregnancy was calculated by subtracting the pre-pregnancy weight (or the weight measured at first appointment, held before 12 weeks) from the weight at delivery (although women had been recruited between 24 and 28 weeks, they were being followed at the Obstetric Clinic and these data were registered in the electronical medical record)

^c^Only the participants with GDM needed insulin. The Mann–Whitney test for independent samples was used to compare continuous variables between the studied groups, while categorical variables were compared by Pearson’s *χ*^2^ test

The Mann-Whitney test *P* ≤ 0.05 was considered significant. The missing data (percentage) for each reported variable is as follows: age (0.77%), ethnicity (8.7%), parity (7.1%), BMI (1.88%), family history (0%), weight gain (4.5%), preeclampsia (0.33%), arterial hypertension (1.2%), smoking (14.6%), use of medicines (1.4%), insulin treatment (0.5%), fasting plasma glucose (5.5%), type of delivery (16.4%), newborn birth weight (16.7%)

Compared to the group without GDM, women with GDM were older, had a higher number of previous pregnancies, a higher pre-pregnancy BMI with a higher frequency of family history of T2D. Women with GDM had less weight gain during pregnancy (probably due to the higher frequency of follow-up visits in comparison with women without GDM; in addition, only the group with GDM attended consultations with dietitians), higher frequency of hypertension and use of medications, and higher FPG compared to women without GDM. Cesarean delivery was more frequent in women with GDM and the weight of newborns from women with GDM was lower than the weight of newborns from women without GDM. Among women with GDM, 18.7% used insulin.

The SNPs rs10187149, rs10194776 and rs765458 did not associate with GDM (Table 2). After binary logistic regression analysis, the minor allele C of SNP rs17619600 conferred an increased risk for GDM in the codominant model (OR 2.15; 95% CI 1.53–3.09; *P* < 0.0001) and in the rare dominant model (OR 2.32; CI 1.61–3.37; *P* < 0.0001). None of the four SNPs were associated with insulin use, maternal weight gain, newborn birth weight or glycemic change in the postpartum OGTT (data not shown).

**Table 2:** Genotype frequencies of single nucleotide polymorphisms in *HTR2B* according to status of gestational diabetes mellitus

SNPs	Without GDM	With GDM	OR (CI 95%)	*P* value
*HTR2B* (N)	443	453		
rs765458				
AA	0.248	0.239	0.90 (0.64–1.27)	0.55 (RD)
AG	0.542	0.518	1.02 (0.82–1.27)	0.82 (CD)
GG	0.210	0.243		
MAF	0.481	0.502		
rs10187149				
AA	0.324	0.338		
AC	0.509	0.498	0.94 (0.77–1.45)	0.70 (RD)
CC	0.167	0.164	0.92 (0.74–1.14)	0.46 (CD)
MAF	0.422	0.413		
rs10194776				
TT	0.277	0.279		
TC	0.526	0.495	0.99 (0.79–1.52)	0.57 (RD)
CC	0.197	0.226	0.96 (0.78–1.19)	0.74 (CD)
MAF	0.479	0.473		
rs17619600				
TT	0.839	0.614	2.32 (1.61–3.37)	< 0.0001 (RD)
TC	0.149	0.329	2.15 (1.53–3.09)	< 0.0001 (CD)
CC	0.012	0.057		
MAF	0.087	0.223		

Analyses were performed in the rare dominant (RD) and in the co-dominant (CD) models after adjustment for age, pre-gestational body mass index and weight gain by binary logistic regression analysis

*CI* confidence interval; *GDM* gestational diabetes mellitus; *MAF* minor allele frequency; *OR* odds ratio; *SNPs* single nucleotide polymorphisms

In evaluating the association between haplotypes in the *HTR2B* gene and the presence of GDM, the selected SNPs were placed in the following order for analysis: rs10194776, rs765458, rs10187149 and rs17619600. The TACC haplotype, which contains the rare allele C of rs17619600, was associated with an increased risk of GDM, as shown in Table 3 (the total number of carriers of the haplotype was 83).

**Table 3:** Frequency of the haplotype TACC in *HTR2B* according to the status of gestational diabetes mellitus

	Without GDM	With GDM	OR (CI 95%)	*P *value
N	443	453		
TACC	60 (0.072)	99 (0.113)	1.70 (1.22–2.39)	0.001

Sequence of single nucleotide polymorphisms: rs10194776, rs765458, rs10187149 and rs17619600. Results expressed in absolute numbers (each carrier with two haplotypes) and relative frequency in the overall population. Haplotype analysis was performed using the software SHEsis

*CI* confidence Interval; *GDM* gestational diabetes mellitus; *OR* odds ratio

**Fig. 1:**
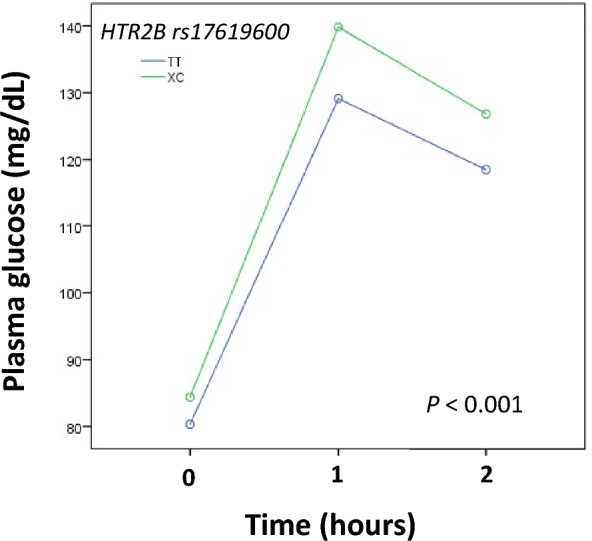
Plasma glucose during oral glucose tolerance test (OGTT) according to genotypes of rs17619600 (rare dominant model). OGTT was performed between 24 and 28 weeks of gestation in 636 women with (*n* = 196) and without (*n* = 440) gestational diabetes mellitus. *P* value was adjusted for age at last menstrual period, previous body mass index, and weight gain during pregnancy

The analysis of plasma glucose during the OGTT performed between 24 and 28 weeks of gestation, for diagnostic purpose, in 636 women with (*n* = 196) and without GDM (*n* = 440) showed that carriers of the XC genotype (rare dominant model) (*n* = 135) presented a significantly higher AUC compared to carriers of the TT genotype (*n* = 501) of rs17619600 (121.52 ± 29.69 *versus* 113.45 ± 24.76; *P* < 0.001) (Fig. 1).

## Discussion

The main finding of the present study was that the presence of the rare allele C in the *HTR2B* rs17619600 SNP conferred an increased risk of GDM in the population evaluated. In addition to the analysis of isolated SNP, the TACC haplotype, which contains the aforementioned allele, was associated with a higher risk of GDM.

*HTR2B* encodes a Gαq-coupled 5-HT receptor. 5-HT is believed to be critical in regulating pancreatic beta cell proliferation [7, 9]. In pregnant rodent islets, there is an increase in the expression of HTR2B during the period of increased beta cell replication; blocking the signaling of this receptor prevents the expansion of these cells and is associated with GDM [8]. In human islets, the activation of this receptor is associated with GSIS [7, 25]. Thus, 5-HT signaling through HTR2B plays an important role in the maintenance of glycemic homeostasis during pregnancy [16, 26, 27].

The only study that evaluated the SNP rs17619600 in *HTR2B* found no association with GDM. However, it was a case–control study which compared women with GDM with non-pregnant women, aged 60 and older with no personal and family history of DM. The study did not find any association of this SNP with weight gain during pregnancy, postpartum BMI, FPG, or fasting insulin concentration in women with GDM. In addition, this variant did not associate with waist circumference and BMI in non-diabetic control subjects and in the independent population cohort from the Korean Genome Epidemiology Study, or with T2D in this same cohort [28].

No functional studies were performed with rs17619600, but according to the GTEx Consortium atlas, this SNP has the potential to be functional, as it has a cis-expression quantitative trait loci (eQTL) effect, that is, it modulates gene expression by influencing its transcription rate [29]. In 7 out of 9 tissues evaluated, the presence of the allele C was associated with a lower expression of the *HTR2B* gene.

Given these findings, we hypothesized that SNP rs17619600 could modulate the expression of the *HTR2B* gene in beta cells. Thus, in presence of the rare allele C, there would be a lower expression of this receptor, which could impair maternal beta cell adaptation. The finding that carriers of the genotypes containing the allele C presented a higher AUC of plasma glucose during OGTT than carriers of the TT genotype corroborates that SNP rs17619600 influences glucose homeostasis, probably affecting insulin release, since activation of HTR2B promotes GSIS.

This study has the limitation of having been carried out in a tertiary hospital, in which a significant number of patients have other comorbidities. The small number of patients who used insulin, only 84 participants, made it difficult to assess the association of SNPs with GDM severity. As GDM is a prevalent clinical condition, the lack of replication in an independent population and the sample size are also limitations of the study, although the present series is larger than those included in several previously published studies [30–34]. Although genetic testing is not yet justified in clinical practice, broadening the spectrum of candidate genes may help to elucidate mechanisms underlying GDM.

## Conclusion

SNP rs17619600 in the *HTR2B* gene influences glucose homeostasis, probably modulating insulin release, and the presence of the minor allele C was associated with a higher risk of GDM.

## Data Availability

The data sets used and analyzed during the current study are available from the corresponding author on reasonable request.

## Abbreviations

5-HT: 5-Hydroxytryptamine
ADA: American Diabetes Association
AUC: Area under the curve
BMI: Body mass index
CI: Confidence interval
DM: Diabetes mellitus
eQTL: Expression quantitative trait loci
FPG: Fasting plasma glucose
GAS: Genetic association study
GDM: Gestational diabetes mellitus
GSIS: Glucose-stimulated insulin secretion
HTR1–7: Serotonin receptor subtypes 1–7
HWE: Hardy–Weinberg equilibrium
IADPSG: International Association of Diabetes and Pregnancy Study Groups
LMP: Last menstrual period
MAF: Minor allele frequency
MTNR1B: Melatonin receptor 1B
OGTT: Oral glucose tolerance test
OR: Odds ratio
PL: Placental lactogen
PPARG: Peroxisome proliferator activated receptor γ
PRLP: Prolactin receptor
RNA: Ribonucleic acid
SNP: Single nucleotide polymorphisms
T2D: Type 2 diabetes mellitus
TCF7L2: Transcription factor 7-like 2
Tph1: Isoform 1 of tryptophan hydroxylase
Tph2: Isoform 2 of tryptophan hydroxylase
